# Oxathiazinane derivatives display both antineoplastic and antibacterial activity: a structure activity study

**DOI:** 10.1007/s00432-023-04799-8

**Published:** 2023-05-12

**Authors:** B. Majchrzak-Stiller, M. Buchholz, I. Peters, J. Strotmann, J. Möhrke, L. Zelichowski, L. Oehlke, C. Quensel, D. Fein, P. Höhn, T. Müller, W. Uhl, C. Braumann

**Affiliations:** 1 Division of Molecular and Clinical Research, Department of General and Visceral Surgery, St. Josef-Hospital, Ruhr-University Bochum, 44791 Bochum, Germany; 2 Geistlich Pharma AG, Wolhusen, Switzerland; 3 Department of General, Visceral and Vascular Surgery, Evangelische Kliniken Gelsenkirchen, Akademisches Lehrkrankenhaus der Universität Duisburg-Essen, Gelsenkirchen, Germany

**Keywords:** Apoptosis, Chemotherapy, Cancer, Substance GP-2250, Reactive oxygen species (ROS)

## Abstract

**Purpose:**

The Oxathiazinane substance class is characterized by a high diversity of chemical structures yet to be fully investigated. Our research group recently proved that the 1.4.5-oxathiazine-4.4-dioxide, known as substance GP-2250, possesses antineoplastic properties as shown on pancreatic carcinoma. This current study aims to gain insights into the structure and activity relationship of a series of different Oxathiazinanes regarding their antineoplastic activity and the potential correlation with antibacterial activity. We investigated the newly synthesized Oxathiazinane derivatives: 2255, 2256, 2287, 2289, 2293 and 2296 in comparison to GP-2250.

**Methods:**

The antineoplastic effect was evaluated in different cancer entities (breast, skin, pancreas and colon cancer cell lines) by viability, proliferation, and cell migration assays in vitro. Disc diffusion tests were performed on various bacteria strains to examine the antibacterial potential. Additionally, reactive oxygen species (ROS) assays were conducted to investigate mechanistic aspects.

**Results:**

The substances GP-2250, 2293, 2289 and 2296 not only showed antineoplastic activity in four different cancer entities but also antibacterial effects, as tested on multiple bacteria strains including MRSA (Methicillin-resistant *Staphylococcus aureus*). Furthermore, these substances also induced high ROS levels up to 110% in the treated cancer cell lines compared to untreated control cells. These results indicate a correlation between an antineoplastic capacity and antibacterial properties of these derivatives. Both activities appear to be ROS driven. The Oxathiazinane derivatives 2255, 2256 and 2287 lacked both, antineoplastic and antibacterial activity.

**Conclusion:**

Thus, a comparable structure activity relationship became apparent for both the antineoplastic and antibacterial activity.

**Supplementary Information:**

The online version contains supplementary material available at 10.1007/s00432-023-04799-8.

## Introduction

The development of efficient cancer treatment remains one of the biggest challenges in modern medicine. Cancer remains among the leading causes of death with 89.6 million cases in 2018 (World Health Organization 2020). While incidence is projected to increase over the next 20 years, therapeutic success remains poor (Janssens et al. 2018; Le Saux and Falandry 2018). Especially for certain cancer entities, like pancreatic cancer or triple negative breast cancer, no convincing therapeutic options are currently available (World Health Organization 2020). This illustrates the importance of discovering new effective agents in modern cancer research.

**Fig. 1:**
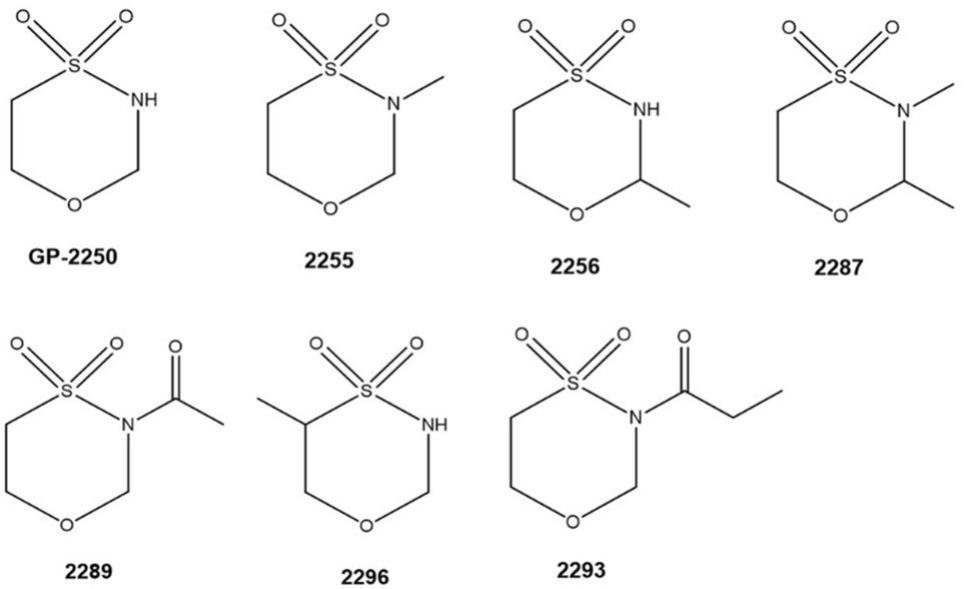
Chemical structures of all tested derivatives

Oxathiazinane derivatives represent a heterogenic substance class encompassing compounds characterized by an aliphatic cyclic six-membered ring structure containing one heteroatom of oxygen, nitrogen and sulfur (Fig. 1). The properties of the compounds belonging to this substance class are highly diverse and have only been rarely investigated (Munck et al. 2015; Qian et al. 2016). Recently, 1.4.5-Oxathiazinane derivatives came into focus in oncological research, especially with the tetrahydro-1.4.5-oxathiazine-4.4-dioxide, known as substance GP-2250 (Fig. 1) (Buchholz et al. 2017).

GP-2250 shows highly antineoplastic capacities (Buchholz et al. 2017) presenting a new therapeutic option in anti-cancer treatment. Our group was the first to investigate the antineoplastic effects of substance GP-2250 on malignant pancreatic cancer in vitro and in vivo. Our study revealed extensive antineoplastic properties of substance GP-2250 in a dose response relationship mainly driven by reactive oxygen species (ROS). In vivo, GP-2250 effectively inhibited pancreatic cancer tumor growth in a murine model of patient derived xenografts as well as in a xenograft derived from established cancer cell lines. The substance showed a very low toxicity profile with an acute maximal tolerable dose of 1500 mg/kg*body weight (Buchholz et al. 2017).

Since the chemical features of Oxathiazinane derivatives are diverse, small changes in structure may have strong impacts in their properties. The specific mechanism of action of tetrahydro-1.4.5-oxathiazine-4.4-dioxide GP-2250 is still subject to current research.

This study focusses on two aspects: 1. The assessment of the antineoplastic properties of chemically distinct Oxathiazinane derivatives in various cancer cell lines using viability, proliferation and migration testing (BrdU, MTT and scratch assay). 2. The assessment of the potential antibacterial properties of these derivatives. This was based on the finding that several established anti-tumor agents such as the anthracycline Doxorubicine (Minotti et al. 2004), the actinomycine Dactinomycin (Cai et al. 2016), the camptothecin Irinotecan (Pommier et al. 2010) and various Bleomycins (Chen et al. 2008) are also known to have antibiotic properties. The potential antibacterial capacities of Oxathiazinane derivatives are yet unknown. It was therefore tested whether a correlation exists between antineoplastic and antibacterial activity of the test compounds, the antibacterial activity was assessed by a disc diffusion test. Thus, this study aims to elucidate a structure activity relationship for the antineoplastic and antibacterial capacities of a series of oxathiazine derivatives.

## Materials and methods

### Disc diffusion test

The antibacterial potential of all Oxathiazinane derivatives were examined by the Kirby-Bauer disc diffusion test using Müller–Hinton agar. A 10 mm diameter disk soaked in 150 µl of distilled water containing 10 mg of the test substance (60 mg/mL) was placed on Müller-Hinton agar (bioMeieux, Genève, Switzerland) holding a pre-lawn of the according test bacteria (*Enterococcus faecium* (vancomycin-resistant Enterococcus, VRE), *Staphylococcus aureus* ATCC 6538, *methicillin-resistant Staphylococcus aureus* (MRSA), *Clostridium difficile, Acinetobacter baumannii*, *Pseudomonas aeruginosa*, *Stenotrophomonas maltophilia, Citrobacter freundii*, *Escherichia coli* ATCC 8739, *Escherichia coli* (extended spectrum beta-lactamase, ESBL), *Klebsiella pneomoniae* (ESBL), *Morganella morganii*, *Bacteroides fragilis*, and *Helicobacter pylori*). The plates were incubated at 37 °C for 24 h and the zone of inhibition was recorded. Culture media and sterilized distilled water were used as a control. *N*-acetylcysteine (NAC) was used as an antioxidant control. A zone of inhibition > 10 mm was considered a positive result.

### Reagents

The powdered Oxathiazinane derivatives 2244, 2250, 2255, 2256, 2287, 2289, 2293 and 2296 (kindly provided by Geistlich Pharma AG, Wohlhusen, Switzerland) and *N*-acetyl-l-cysteine (Sigma Aldrich, Taufkirchen, Germany) were dissolved in double distilled water (ddH_2_O), adjusted to a physiological pH and sterile filtered before use. Cisplatin (CisP, Hexal AG, Holzkirchen, Germany) and nabPaclitaxel (Abraxane) (Celgene Corp., Summit USA) were dissolved according to manufactures instructions in 0.9% sodium chloride and set to a physiological pH by the St. Josef-Hospital Bochum pharmacy and was stored protected from light at 4 °C.

### Cell lines and culture conditions

Four different human cancer cell lines representing four major cancer types (pancreatic cancer, colon cancer, breast cancer and skin cancer) were used for our experiments: pancreatic cancer cells Panc Tu I (CLS Cell Lines Service, Eppenheim, Germany), colon cancer cells HCT116 (ATCC-LGC Standards GmbH, Wesel, Germany), merkel cell carcinoma cells MCC 14.2 and mammary gland/breast-cancer cells MDA MB 468 (ATCC-LGC Standards GmbH, Wesel, Germany). All cell lines were passaged less than 6 months and authentication was performed by STR analysis. HCT 116, MDA MB 468 and Panc Tu I cells were cultured in Dulbecco´s Modified Eagle Medium (DMEM), MCC 14.2 was maintained in RPMI 1640. All cultures were supplemented with penicillin (100 U/ml), streptomycin (100 U/ml) and 2 mM l-Glutamine. MCC 14.2 cells were further complemented with 25 mM HEPES. Cells were grown as monolayer at 37 °C and 5% CO_2_ in a humidified atmosphere.

Different gram-negative and gram-positive bacteria were used for our experiments: *Staphylococcus aureus*, *methicillin-resistant Staphylococcus aureus* (MRSA) and *Escherichia coli* (ATCC – LGC Standards GmbH, Wesel, Germany); *Enterococcus faecalis*, *Enterococcus faecium, Staphylococcus aureus*, *Clostridium difficile*, *Acinetobacter baumannii*, *Pseudomonas aeruginosa*, *Stenotrophomonas maltophilia*, *Citrobacter freundii*, *Escherichia coli* (*E. coli*), *Klebsiella pneomoniae, Morganella morganii*, *Bacteroides fragilis*, *Helicobacter pylori* (fresh clinical isolates). All isolates were cultured with Müller-Hinton-Agar excluding *Bacteroides fragilis* and *Helicobacter pylori*. *Bacteroides fragilis* was cultured with Müller-Hinton-Agar containing 5% horseblood and 20 mg/l ß-NAD. *Helicobacter pylori* was cultured with Chocolate PolyViteX-Agar.

### MTT cytotoxicity assay

Cells were seeded individually in 100 µl to obtain a subconfluent monolayer (HCT 116, Panc Tu I and MCC 14.2 15.000 cells, MDA MB 468 20. 000 cells) in a 96-well plate format and were incubated for 24 h prior treatment. To examine a dose response regarding the antineoplastic activity, cells were incubated with increasing concentrations (50, 100, 200, 300, 400, 500, 750, 1000, 1500, 2000 µM) of each Oxathiazinane derivative and ddH_2_O as control for 24 h. Following stated exposition times, 10 µl MTT (3-(4,5-Dimethylthiazol-2-yl)-2,5-diphenyltetrazoliumbromid) reagent (5 mg/ml) was added and incubated for 2 h before violet Formazan crystals were dissolved in 100 µl DMSO (Dimethylsulfoxide). Viability of the cells was analyzed via microplate absorbance reader by measuring the optical density at a wavelength of 560 nm (reference wavelength 720 nm) (ASYS, UVM340, Anthos Mikrosysteme GmbH, Germany). The assay was performed using 8 replicates in three independent experiments with consecutive passages.

### BrdU proliferation assay

Cells were seeded individually in 100 µl to obtain a subconfluent monolayer (HCT 116, Panc Tu I and MCC 14.2 15.000 cells, MDA MB 468 20. 000 cells) in a 96-well plate format and incubated for 24 h prior treatment. To examine the dose response regarding their anti-proliferative activity cells were incubated with increasing concentrations (100, 300, 500 and 2000 µM) of each Oxathiazinane derivate and ddH2O as control for 6 h prior BrdU proliferation assay (5-bromo-2-deoxyuridine)-ELISA (Roche Applied Science, Mannheim, Germany) according to the manufacturer’s instructions. The incubation time of 6 h has been shown to be appropriate for the BrdU proliferation assay in previous experiments. The amount of synthesized DNA was detected using a microplate absorbance reader measuring at 370 nm with a reference wavelength of 492 nm (ASYS, UVM340, Anthos Mikrosysteme GmbH, Krefeld, Germany). BrdU assays were performed with 8 replicates of three independent experiments with consecutive passages.

### Scratch assay

Cells (Panc TU I, HCT116, MCC 14.2, MDA MB 468) were plated in two ml of medium into the 60-mm dishes to create a confluent monolayer (500,000 cells) and were incubated for 24 h allowing cells to adhere and spread. The required number of cells for a confluent monolayer depended on the particular cell type. After introducing a scratch in the cell monolayer, mimicking a wound, the area was examined by phase-contrast-microscopy in bright field with a 10 × expansion (Axiocam 305 color, Zeiss). Images were captured at the beginning of the treatment (100 µM or 1000 µM) and at regular intervals during cell migration (6, 12, 24, 48 and 72 h) closing the scratch, to semi-quantify the migration rate of the cells. The width of the scratch was measured with the software tool ZEN 3.0 blue edition.

### ROS analysis

To get further insights into functional aspects, the impact of GP-2250, 2289, 2293 and 2296 on cellular ROS level was analyzed. Cells were seeded individually to obtain a subconfluent monolayer in a 96-well plate format and were incubated for 24 h prior treatment. Cells were incubated with 2000 µM of GP-2250, 2289, 2293 and 2296 for 2 h. Untreated cells served as a control for the basic ROS level of the cells. Measurements were performed using the Cellular ROS/Superoxide Detection Assay KIT (ab139476, Abcam, Cambridge, UK) following the manufacturer’s instructions. Measurements were performed at 490 nm extinction and 520 nm emission. The assay was performed using 4 replicates in three independent experiments with consecutive passages.

### Statistics

Results of MTT and BrdU assay (percentage of living/proliferating cells) are expressed as means ± standard derivation (SD). We used one-way ANOVA followed by Tukey’s post-hoc test comparing experimental groups with normal distribution and Fisher´s exact test for categorical data if appropriate. *P* values ≤ 0.05 were considered as statistically significant and indicated in the figures as follows: ***p ≤ 0.001, **p ≤ 0.01, *p ≤ 0.05.

## Results

### GP-2250, 2289, 2293 and 2296 show a cytotoxic and anti-proliferative effect on all tested cell lines

**Fig. 2:**
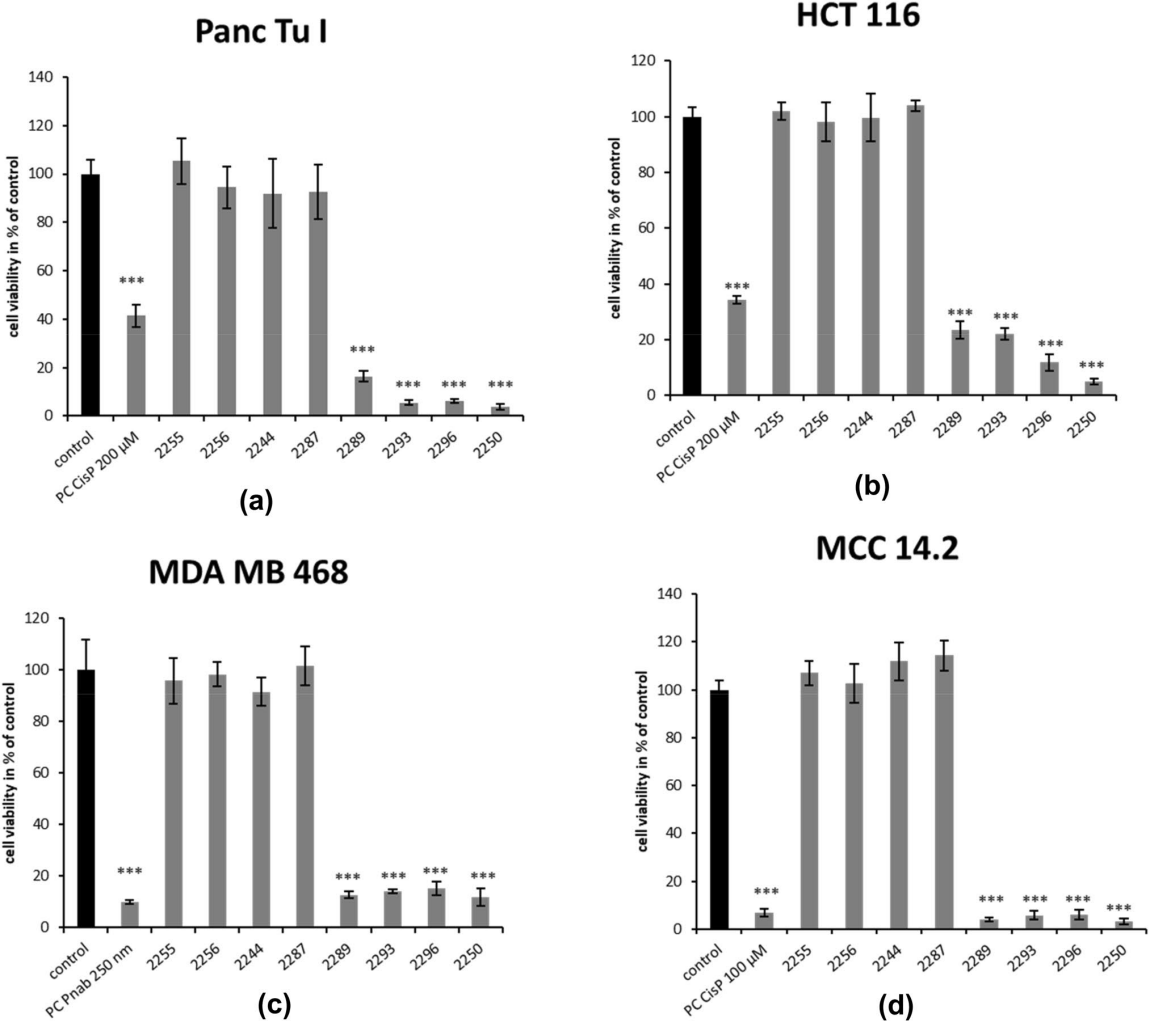
Effects of different Oxathiazinane derivatives in four malignant cell lines measured by MTT-assay. MDA MB 468, Panc Tu I, HCT 116 and MCC 14.2 cells were incubated with 2000 µM of all tested Oxathiazinane derivatives (GP-2250, 2255, 2256, 2287, 2289, 2293, 2296 and PC (Cisplatin or nabPaclitaxel) and ddH_2_O (control) for 24 h and submitted to an MTT-assay. Values are means ± SD of 8 replicates of three independent experiments with consecutive passages. Asterisk symbols indicate differences between control, which was adjusted to 100% and treatment. ***p ≤ 0.001, **p ≤ 0.01, *p ≤ 0.05, n.s. *p* > 0.05 (one-way ANOVA followed by Tukey’s post-hoc test). Incubation with a concentration of 2000 µM for 24 h with GP-2250, 2289, 2293 and 2296 resulted in a significant reduction of living cells within all tested cancer cell lines similar to the accompanied positive control. All other tested derivatives showed no reduction in cell viability with all tested cancer cell lines

We first determined which of the derivatives shared the antineoplastic activity with GP-2250. The effect of all derivatives on cell viability and cell proliferation was tested in a cell culture model (MTT tests and BrdU assays) using four different cancer cell lines (pancreas-, colon-, merkel cell- and breast cancer). As positive controls, a standard chemotherapeutic agent relevant for the individual cancer entities were used (Cisplatin or nabPaclitaxel). In the first step a single rather high dose of 2000 µM was used in the present study for all compounds to detect even minor effects and to distinguish clearly between effective and ineffective substances. The MTT-assay results presented in Fig. 2 for all derivatives demonstrate a distinct reduction of living cells within all tested cancer cell lines compared to untreated controls (equivalent to 100%) after 24-h incubation with 2000 µM of each test compound (GP-2250, 2289, 2293, 2296, 2255, 2256, 2287).

All four different cancer entities showed high susceptibility towards GP-2250, 2289, 2293 and 2296 with response rates over 80%. GP-2250 inhibited cell viability most effectively resulting in a reduction of cell viability by over 90%. The compounds 2289, 2293 and 2296 similarly decreased cell viability in all cell lines resulting in fewer than 20% viability in treated cultures with exception of HCT 116, which showed a slightly lower effectiveness amounting to a reduction in cell viabilities to 23.4% for 2289 and 22.1% for 2293.

In contrast to this pronounced reduction in cell viability, the remaining compounds (2255, 2256, and 2287) failed to reduce cell viability in all four cancer cell lines tested at 2000 µM. Thus, there seems to be a pronounced structure activity relationship in cytotoxicity among the oxathiazines tested (Fig. 2). These findings were supported by the similar results on the impact on the proliferation of cancer cells, using BrdU assays, as described below.

To be able to better classify the effectiveness of the active substances, MTT assays with rising concentrations were performed to determined the EC 50 of the active substances (Figure S1). The lowest EC 50 values were shown with GP-2250 in all analyzed cell lines (Panc TuI 589 µM, HCT 116 423 µM, MCC 14.2 419 µM and MDA MB 468 89 3 µM) followed by 2289 and 2296 (Table 1). Substance 2293 was less effective with EC 50 values of 977 µM in Panc TuI, 1108 µM in HCT 116, 558 µM in MCC 14.2 and 1489 µM in MDA MB 468.

**Table 1:** EC 50 of the effective substances

	Substance	EC 50 [µM]
HCT 116	2250	423
	2289	867
	2293	1108
	2296	810
MCC 14.2	2250	419
	2289	549
	2293	558
	2296	556
Panc TUI	2250	589
	2289	939
	2293	977
	2296	706
MDA MB 468	2250	893
	2289	1214
	2293	1489
	2296	1164

**Fig. 3:**
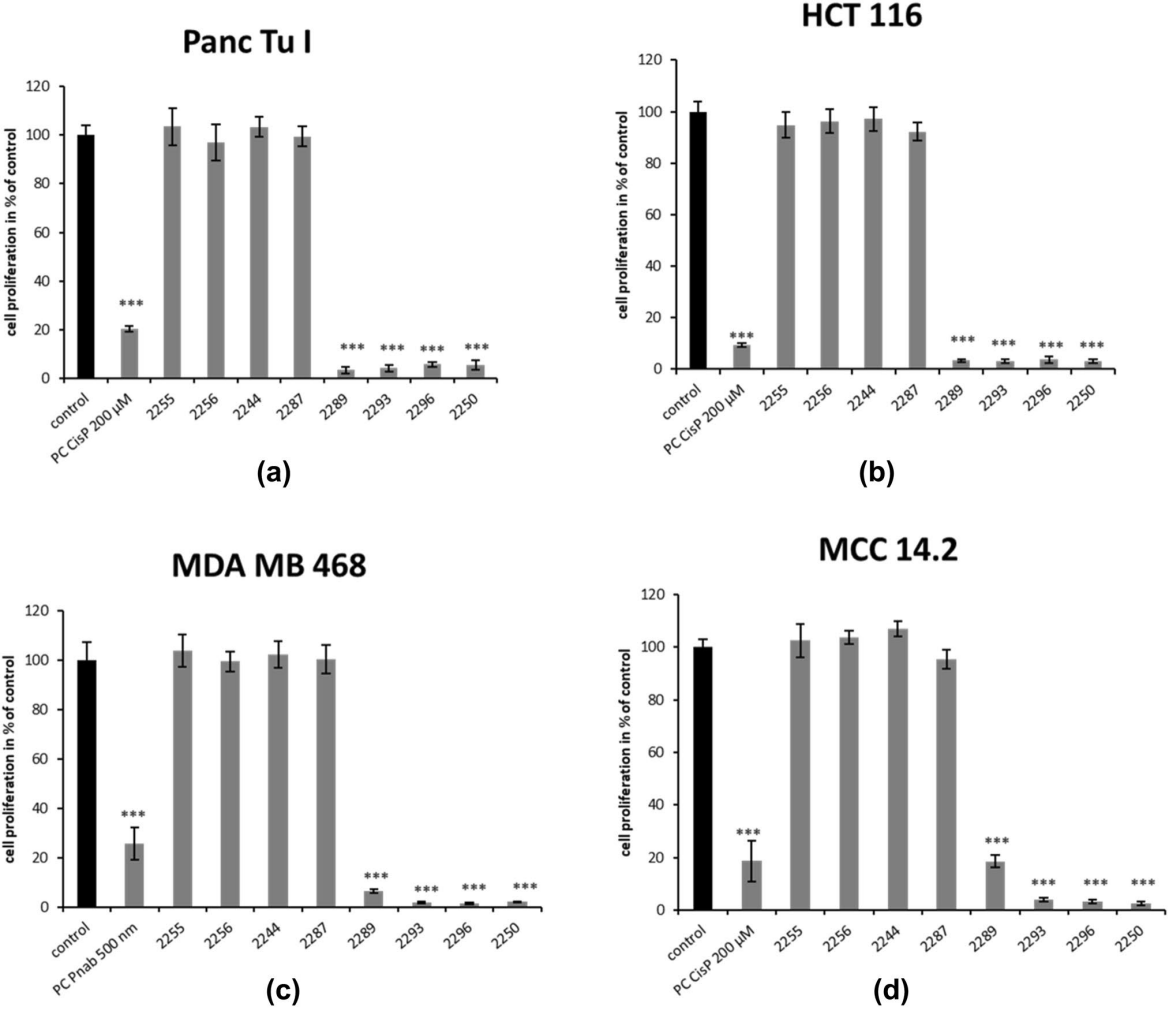
Effects of different Oxathiazinane derivatives on cell proliferation in four malignant cell lines measured by BrdU assay. MDA MB 468, Panc Tu I, HCT 116 and MCC 14.2 cells were incubated with 2000 µM of the different Oxathiazinane derivatives (GP-2250, 2255, 2256, 2287 2289, 2293, 2296) and positive control (PC) Cisplatin (CisP) or nabPaclitaxel (Pnab) and ddH_2_O (control) for 6 h and submitted to a BrdU assay. Values are means ± SD of 8 replicates of three independent experiments with consecutive passages. Asterisk symbols on columns indicate differences between control, which was adjusted to 100% and GP-2250 treatment. ***p ≤ 0.001, **p ≤ 0.01, *p ≤ 0.05, n.s. *p* > 0.05 (one-way ANOVA followed by Tukey’s post-hoc test). Incubation with 2000 µM of GP-2250, 2289, 2293 and 2296 for 6 h resulted overall in a significantly reduced proliferation rate of all analyzed cell lines like the accompanied positive control. All other derivatives showed no effect within all tested cancer cell lines

The anti-proliferative effect of all derivatives was analyzed via BrdU assay applying the same concentrations used for the MTT test. Overall incubation with 2000 µM of GP-2250, 2289, 2293 and 2296 for 6 h resulted in a significant reduction of cell proliferation, whereas the remaining derivatives showed no effect within all tested cancer cell lines. GP-2250 inhibited proliferation most effectively, leading to values of the remaining proliferating cells ranging between (2.1%) in MDA MB 468 and (5.4%) Panc Tu I (Fig. 3).

This data indicates a significant cytotoxic effect of substances GP-2250, 2289, 2293 and 2296 on all analyzed cell lines resulting in fewer than 10% proliferation in all treated cultures. Only MCC 14.2 treated with 2000 µM 2289 showed a remaining cell proliferation of 18.6% respectively.

In summary, GP-2250, 2289, 2293 and 2296 displayed significant cytotoxic and growth inhibitory effects resulting in major impacts on the cell viability and cell proliferation on all cancer cell lines. In contrast, no significant reduction in either cell viability or cell proliferation was observed after treatment with the compounds 2255, 2256, and 2287 at 2000 µM (Fig. 3).

Comparable with the MTT assay, a BrdU assay with rising cancentrations of the active substances was performed. The results were similar to the previous tests. GP-2250 showed the highest inhibition rate in all analyzed cell lines, followed by the other three compounds, which show slightly variable effects in the analyzed cell lines (Figure S2).

### GP-2250, 2289, 2293 and 2296 present a migration inhibiting effect on all cancer cell lines

The migration rate of all examined cancer cell entities (pancreas-, colon-, merkelcell- and breast cancer) was quantified using scratch-assays under treatment with all test compounds for 48 h to 5 days. As positive controls, standard chemotherapeutic agents relevant for the individual cancer entities were applied.

**Fig. 4:**
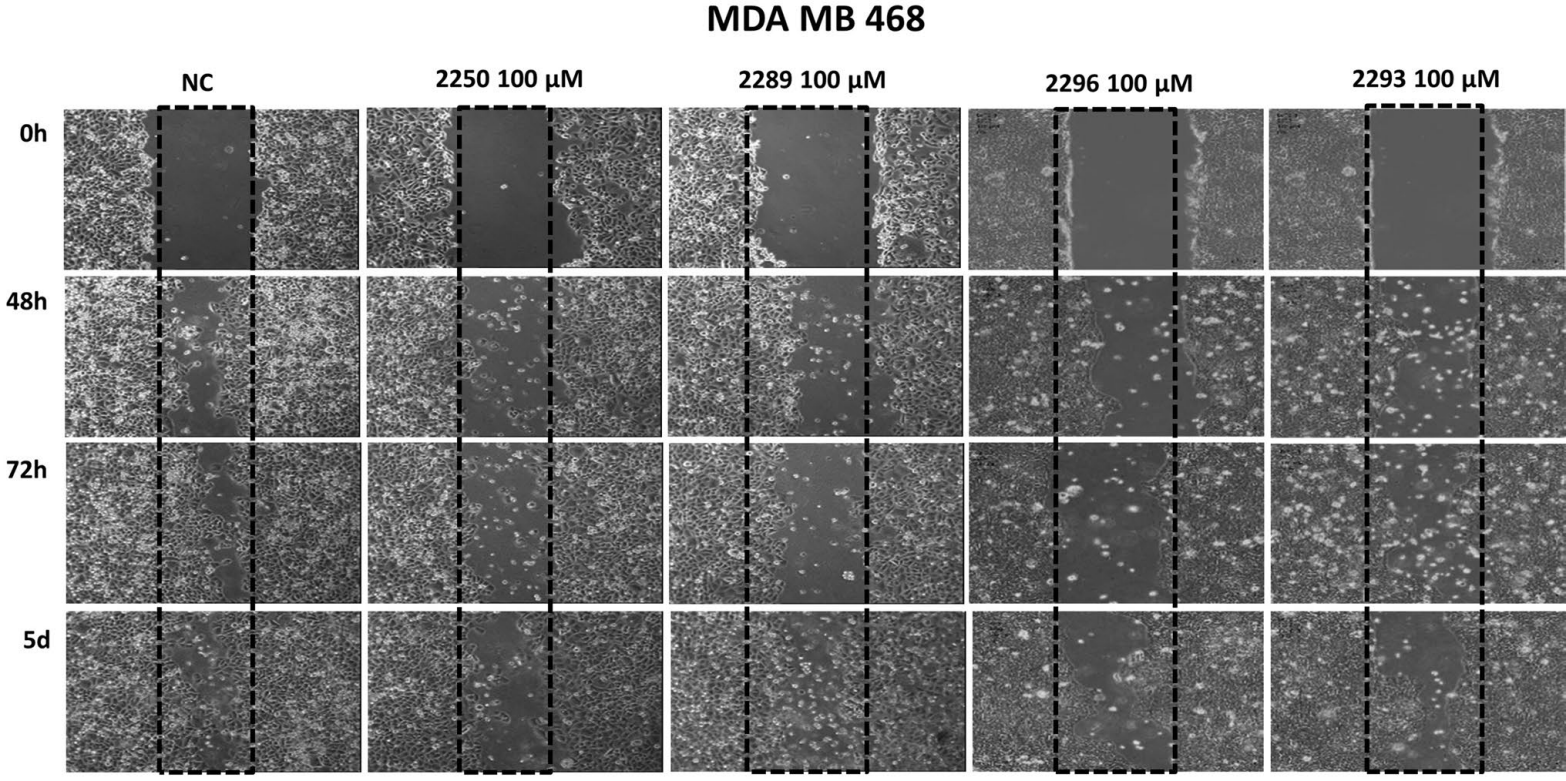
Histological results of the migration assay of different Oxathiazinane derivatives treated for 48 h to 5 days illustrated exemplary using a confluent monolayer of MDA MB 468. Compared to the untreated control (NC) the migration rate in cultures treated with GP-2250, 2289, 2293, 2296 (100 µM) were clearly inhibited. The tested compounds 2255, 2256 and 2287 (1000 µM) showed no reduced migration rate, yielding nearly completely overgrown scratches after 5 days (data not shown). These results are representative for all tested cell lines

Figure 4 displays the results exemplary illustrating the test with a confluent monolayer of MDA MB 468. The results are representative for all analyzed cell lines. All components were compared to an untreated control (NC) where the cells migrated far into the scratch area to close the gap after an incubation period of 5 days. In contrast, the migration rate in cultures treated with GP-2250, 2289, 2293 and 2296 was markedly inhibited. The effective components GP-2250 as well as 2293 and 2296 displayed a slightly stronger anti-migratory effect on the cells than 2289, resulting in more pronounced gaps after an incubation period of 5 days. In MDA MB 468, a concentration of 100 µM of test compounds was used due to the intrinsic low migration rate. In all other cell lines, a concentration of 1000 µM was used.

**Fig. 5:**
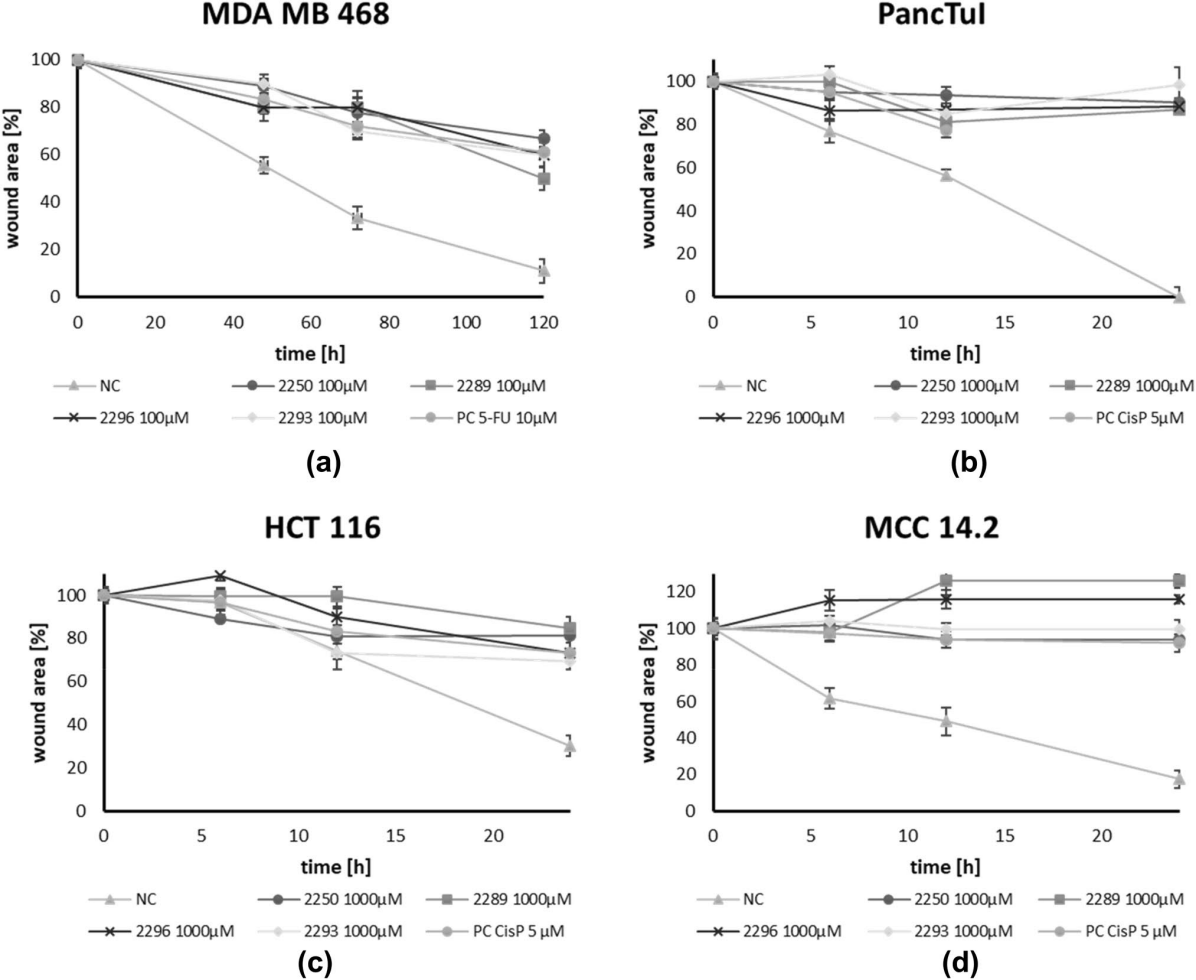
Results of the migration assay of different Oxathiazinane derivatives in % of wound area, for **a** PancTuI, **b** HTC 116, **c** MDA MB 468, **d** MCC 14.2. Compared to the untreated control (NC) the wound healing in cultures treated with GP-2250, 2289, 2293, 2296 (100 µM for MDA MB 468 and 1000 µM for all other cell lines) was clearly inhibited in a time dependent manner. Positive control PC: CisP or 5-FU. The tested compounds 2255, 2256 and 2287 (1000 µM) showed no reduced wound healing (S3). Values are means ± SD of three independent experiments with consecutive passages

However, cell cultures treated with 2255, 2256 and 2287 at the concentration of 1000 µM, displayed no reduced migration rate. Here, gaps were almost completely closed after 5 days, analogously to the control. Figure 5 summarizes and quantifies all results of the migration assays within all four cancer entities. Compared to the untreated control (NC) the wound healing in cultures treated with GP-2250, 2289, 2293, 2296 was clearly inhibited in a time dependent manner in all cancer cell lines. In contrast, the compounds, 2255, 2256 and 2287 (1000 µM) showed no reduced wound healing (Figure S3).

### 2289, 2293 and* 2296 show an impact on reactive oxygen species (ROS) comparable to GP-2250*

The impact of GP-2250 on the intrinsic cellular ROS levels of tumor cells has been shown in previous studies [6]. Based on structural similarities, it was of interest to test the ROS levels following treatment with the four oxathiazine derivatives which had shown antineoplastic activity in the former experiments in all four cancer cell lines.

**Fig. 6:**
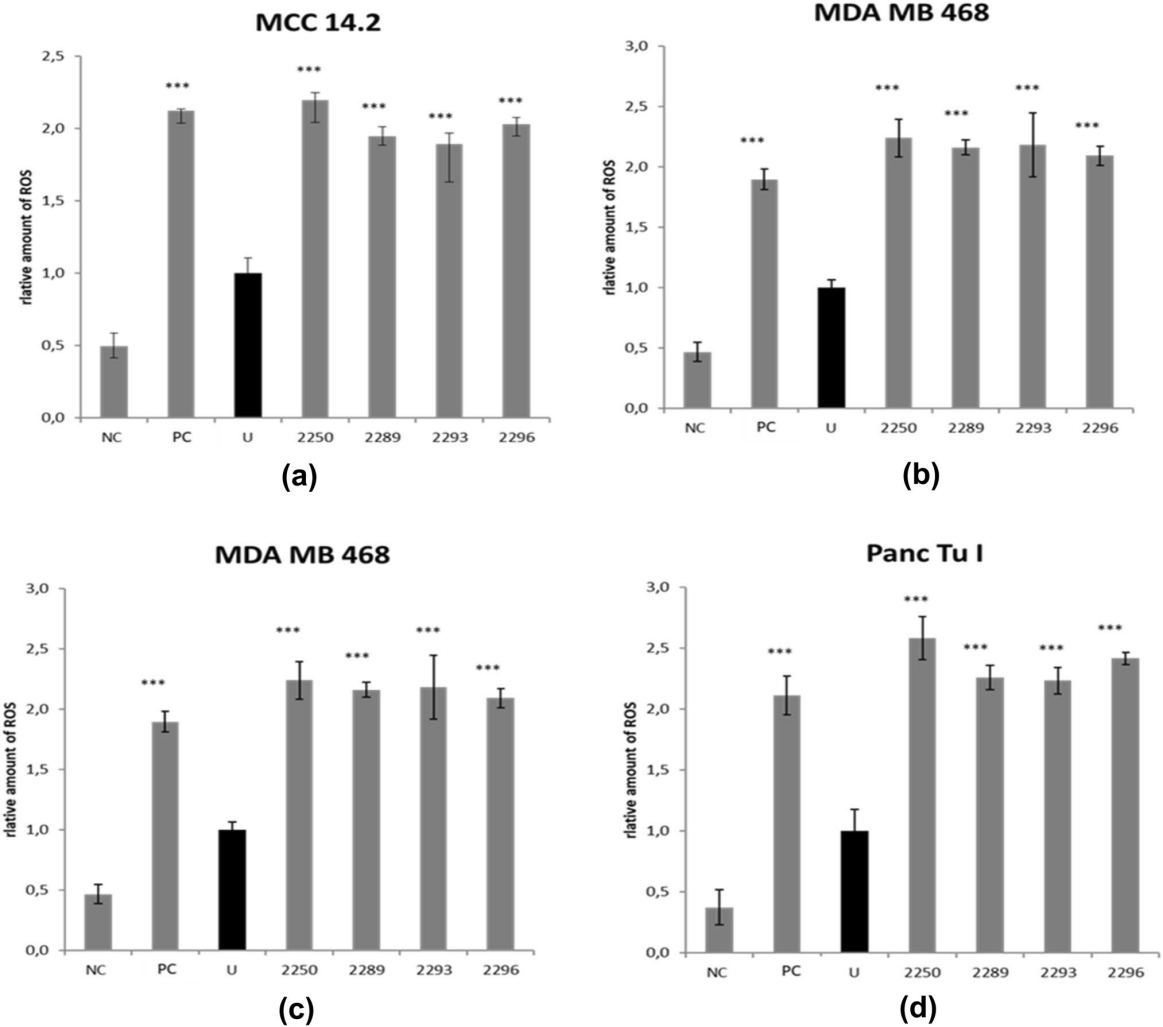
Results obtained for the impact of GP-2250, 2293, 2296 and 2289 on the cellular level of ROS. MDA MB 468, Panc Tu I, HCT 119 and MCC 14.2 cells were incubated with either GP-2250, 2293, 2296 and 2289 (2000 µM), or ddH_2_O as control for 1 h and the level of ROS was analyzed. Additional NAC treatment served as a negative control. Positive Control (PC) was provided by the kit. Values are means ± SD of 4 replicates of three independent experiments with consecutive passages. Asterisk symbols indicate differences between untreated control (U), which was adjusted to 1, and treatment. Negative Control (NC) contained GP-2250 (2000 µM) and 5 mM NAC provided with the kit. ***p ≤ 0.001, **p ≤ 0.01, *p ≤ 0.05 (one-way ANOVA followed by Tukey’s post-hoc test). Data from these two assays clearly show that all effective substances as well as GP-2250 act via a redox-directed mechanism independent of the type of malignant tumor cell line

All oxathiazine derivatives with antineoplastic potential, including GP-2250, displayed increased amounts of ROS in all cancer entities compared to untreated controls, as illustrated by Fig. 6. Cells treated with the radical scavenger N-acetylcysteine (NAC) as negative control) showed diminished amounts of oxygen species after treatment (Fig. 6).

### GP-2250 2289, 2293 and 2296 show antibacterial potential towards various bacteria including MRSA

It was of interest to test whether the structure activity relationship identified for the antineoplastic activity of the Oxathiazinanes would include an antbacterial activity. To analyze the effect of all Oxathiazinane derivatives Kirby–Bauer disc diffusion tests were performed using different bacterial strains including *methicillin-resistant Staphylococcus aureus* (MRSA). Table 2 presents the results obtained from the disc diffusion test evaluating the antibacterial properties of all Oxathiazinane derivatives. Notably, only substances GP-2250, 2289, 2293 and 2296 show a significant impact on the viability of all tested bacteria. Especially, the effect on MRSA (27.5–49.5 mm) should be particularly highlighted (Table 2). The remaining Oxathiazinane derivatives did not effectively inhibit bacterial growth within this experimental setting of the Kirby–Bauer disc diffusion test, presenting with inhibition zones of less than 10 mm. Thus, no antibacterial effect can be attributed to the derivatives 2255, 2256 and 2287.

**Table 2:** Results obtained from the disc diffusion test analyzing the antibacterial properties of all Oxathiazinane derivatives

Substance	Zone of Inhibition	Bacteria
2250	+ (24.5 mm; 49.5 mm; 39,0 mm, 35.0 mm, 16.0 mm, 66.5 mm)	Various a.o. *Escherichia coli*, *Staphylococcus aureus* (MRSA), *Enterococcus faecium (VRE), Acinetobacter baumannii, Pseudomonas aeruginosa, Helicobacter pylori*
2255	− (< 10 mm)	Various a.o. *Escherichia coli*, *Staphylococcus aureus* (MRSA)
2256	− (< 10 mm)	*Escherichia coli*, *Staphylococcus aureus* (MRSA)
2287	− (< 10 mm)	*Escherichia coli*, *Staphylococcus aureus* (MRSA)
2289	+ (18.5 mm; 27.5 mm)	*Escherichia coli*, *Staphylococcus aureus* (MRSA)
2293	+ (19.5 mm; 31.5 mm; 32.5 mm)	*Escherichia coli*, *Staphylococcus aureus*, *Staphylococcus aureus* (MRSA)
2296	+ (23.0 mm; 38.0 mm; 43.0 mm)	*Escherichia coli*, *Staphylococcus aureus*, *Staphylococcus aureus* (MRSA)

GP-2250 as well as 2289, 2293 and 2296 (60 mg/mL) show a significant impact on the viability of all tested bacterial strains, which was most pronounced for MRSA (up to 66.5 mm zone of inhibition by GP-2250)

### The antibacterial effect of GP-2250 is inhibited by a radical scavenger

As previously described, the pro-oxidant mechanism mediating antineoplastic action of GP-2250 is inhibited in the presence of the radical oxygen scavenger N-acetylcysteine (NAC) [6]. Likewise, the addition of NAC inhibited the antibacterial activity of GP-2250 (Table 3). This suggests a common mechanism enabling GP-2250 to impair cell viability, not only in tumor cells but also in bacteria. This result is especially noteworthy as the GP-2250 sensitive bacterial strains include MRSA.

**Table 3:** Results obtained from the agar diffusion test analyzing the inhibitory effect of *N*-acetylcysteine (NAC)

Substance	N-acetylcysteine	Zone of Inhibition	Bacteria
GP-2250	0 mg	24.5 mm	*E. coli*
GP-2250	1 mg	19.5 mm	*E. coli*
GP-2250	2 mg	16.0 mm	*E. coli*
GP-2250	5 mg	14.5 mm	*E. coli*

With increasing concentration, the radical scavenger NAC inhibited the antibacterial effect of substance GP-2250

### Common structure activity relationship for antineoplastic and antibacterial actions

In summary, all results indicate that substance GP-2250 as well as the derivatives 2289, 2293 and 2296 possess pronounced antineoplastic activity, expressed by effective inhibition of cell viability, cell proliferation and cell migration of cancer cells from four cancer entities (pancreas-, colon-, merkel cell- and breast cancer). The remaining derivatives 2255, 2256 and 2287 proved to yield no antineoplastic activity (Figs. 2, 3,4, 5). Similarly, GP-2250, 2289, 2293 and 2296 displayed antibacterial activity, most notably against MRSA, while the Oxathiazinanes 2255, 2256, and 2287 failed to show antibacterial activity (Table 2). These results demonstrate a unifying structure activity relationship (Table 4). The increase of ROS in cancer cells may point to common mechanistic determinants. As both, the antineoplastic activity and the antibacterial activity could be diminished by the addition of NAC (Table 3), the Oxathiazinanes are likely to act by exacerbating ROS-driven cellular damage.

**Table 4:** Overview of the results obtained for the antineoplastic and antibacterial assays

Substance	Antibacterial capacity	Antineoplastic capacity
2250	+	+ +
2255	−	−
2256	−	−
2287	−	−
2289	+	+
2293	+	+
2296	+	+

Substance GP-2250, 2289, 2293 and 2296 indicated promising antineoplastic and antibacterial capacities on all analyzed cultures. All other derivatives can be pronounced as broadly ineffective within the experimental settings, +  +  = EC50 (24 h) < 500 µM min. 3 of four cell lines, +  = EC50 (24 h) 500–1500 µM min. three of four cell lines, − EC50 (24 h) > 1500 µM min three of four cell lines

## Discussion

This study was performed to assess the structure activity relationship of the antineoplastic and potential antibacterial effects of a series of the Oxathiazinane derivatives. For the first time, an antibacterial activity could be demonstrated for GP-2250 and three other derivatives, 2289, 2293 and 2296, with special importance concerning methicillin-resistant *Staphylococcus aureus* (MRSA). Additionally, GP-2250 shows high antibacterial capacities against various pathogens like *Acinetobacter baumannii*, *Pseudomonas aeruginosa* and *Helicobacter pylori* which are listed as critical and high priority at the ‘WHO priority pathogens list for R&D of new antibiotics’ (Word Health Organisation 2017). The Oxathiazinanes 2255, 2256 and 2287 failed to show antibacterial activity pointing to a clear structure activity relationship.

Previous data of our research group had demonstrated the antineoplastic effect of substance GP-2250 (Buchholz et al. 2017). The substance exhibited significant antineoplastic capabilities towards pancreatic carcinoma in vitro and in vivo in a dose dependent manner (established cell lines and xenograft cancer models). In this study comparable results were obtained in additional cancer entities. Apart from GP-2250, three other derivatives (2289, 2293 and 2296) similarly decreased cell viability as well as proliferation- and migration rates on four fundamentally distinct cancer entities (breast, colon, pancreas and merkel cell carcinoma, Figs. 2, 3 and 4). Neither 2244, 2255, 2256 nor 2287 showed inhibitory effects on the viability, proliferation, migration of the cancer cells. A direct comparison of the four active structures using MTT and BrDU assays show clearly, that GP-2250 is the most effective compound (EC 50 between 419 and 893 µM) where the other three derivatives 2289, 2293 and 2296 show comparable higher EC 50 values (549–1489 µM) (Table 1, Fig. S1 and S2) against all four cancer entities.

Antineoplastic and antibacterial effects occurred together exclusively with the same derivatives. Both activities show a comparable structure activity relationship. This extraordinary finding may point to a mutual common mechanism contributing to the inhibition of antineoplastic and the antibacterial activity by oxathiazine derivatives.

Considering the chemical structures of all effective derivatives, 2289 and 2293 possess an additional acetyl- and propionyl-group, respectively, at the nitrogen atom at position 5 of the aliphatic six-membered ring structure. In contrast substance 2296 has an additional methyl-group at the C3 of the six-membered ring structure whereas substance GP-2250 consists only of the aliphatic six-membered ring structure (see Fig. 1).

Methylation of this aliphatic six-membered ring structure at the positions 5 and/or 6 resulted in a complete loss of antineoplastic and anti-infective activities of these GP-2250 derivatives (Fig. 1). As all effective structures possess similar capacities, a common mechanism of action based on the chemical structure has to be assumed but cannot be entirely explained, yet.

As previously demonstrated, ROS-driven programmed cell death is a crucial part of the mechanism of action of GP-2250 in pancreatic carcinoma. Increased levels of ROS are integral in programmed cell death (Galadari et al. 2017), encompassing crucial pathways for apoptosis, autophagy and necroptosis. Concerning apoptosis, ROS is causing a down-regulation of c-FLIP and thus facilitating the extrinsic pathway on the one hand (Wang et al. 2008) but is also triggering the intrinsic pathway through ROS-induced permeabilization of the mitochondric membrane (Galadari et al. 2017). Furthermore, ROS modulates various pathways leading to autophagy (He and Klionsky 2009) and necroptosis (Dixon and Stockwell 2014; Kim et al. 2007). As tumor cells exhibit intrinsically higher ROS levels, these mechanisms are of interest in anti-tumor therapy. Toxic thresholds can be reached in tumor cells while their physiological counterparts are still able to compensate (Nogueira and Hay 2013). Our results show that the Oxathiazinane derivatives of GP-2250, 2289 and 2293 as well as 2296 had indeed the ability to reliably induce ROS to drive cell death in all cancer cell lines of different cancer entities.

N-acetylcysteine is an important antioxidant and a recognized research tool for investigating ROS-induced apoptosis. Its free radical scavenging properties are based on an increase of intracellular glutathione levels and reducing properties (Sun 2010). Consequently, the excessive accumulation of ROS induced by substance GP-2250 in pancreatic cancer cell lines was completely neutralized by the radical scavenger NAC, thereby inducing a broad protection against substance GP-2250 (Buchholz et al. 2017). A similar NAC-sensitivity of antineoplastic action is found for chemotherapeutic platin derivatives (Wang et al. 2014).

Apart from antineoplastic effects, ROS have also been found to show antibacterial effects. Treatment with antibiotics, inter alia Aminoglycoside, Quinolones, Rifampicin and Chloramphenicol led to induction of ROS in bacteria (Vatansever et al. 2013). These radicals were proven to either directly damage DNA, lipids and proteins or indirectly damage DNA via oxidation of the deoxynucleotide pool (van Acker and Coenye 2017).

As all active Oxathiazinane derivatives GP-2250, 2289, 2293 and 2296 induce ROS, we assume, that its antibacterial capacities (Fig. 6) can be partially attributed to ROS induction, which extends to otherwise difficult to treat bacteria, including MRSA, ESBL and VRE. These resistant bacteria pose an increasing challenge in modern medicine as (multi) drug resistant stems increase, causing over 33.000 deaths annually in the EU (Cassini et al. 2019). This aspect might be of special interest for clinical studies. Antibacterial properties of established chemotherapeutics have been described, especially for Tobramycin, Mitomycin C, Bleomycin and Actinomycin D. However, their clinical application as antibacterial agents remains limited due to severe toxicity (Domalaon et al. 2019; Müller and Zahn 1977).

Limitation to this study might be the more qualitative design of the experiments. Rather high concentrations (IC 80) were used, to detect even minor effects of all tested derivatives. Nevertheless, a dose dependency in vitro and in vivo has been described for GP-2250 by Buchholz et al. (2017).

## Conclusions

In addition to the previously known GP-2250, three more Oxathiazinane derivatives were identified as anti-cancer agents. Furthermore, four Oxathiazinanes were identified as antibacterial agents for the first time, with a spectrum that includes MRSA and further highly critical bacterial strains. It is most intriguing that the anti-cancer activity and the antibacterial activity is shared by the same compounds, while other Oxathiazinane derivatives failed to be active in both indications. This finding points to a clearcut structure activity relationship among the Oxathiazinanes tested. This duality is suggestive of a common mechanistic denominator for both activities. So far, the increase in ROS has been identified as a common mechanism affecting the viability of cancer cells and of bacteria. This forms the basis for further mechanistic and in vivo studies.

## Supplementary Information

Below is the link to the electronic supplementary material.

Supplementary file1 (JPG 571 KB)

Supplementary file2 (JPG 442 KB)

Supplementary file3 (JPG 359 KB)

## Data Availability

The data presented in this study are available on request from the corresponding author. The data are not publicly available due to patent legal issues.
