# Supplementary figures and images for: Oxathiazinane derivatives display both antineoplastic and antibacterial activity: a structure activity study
Source: J Cancer Res Clin Oncol. 2023 May 12;149(11):9071–83. doi: 10.1007/s00432-023-04799-8 (PMC10374762; doi:10.1007/s00432-023-04799-8)

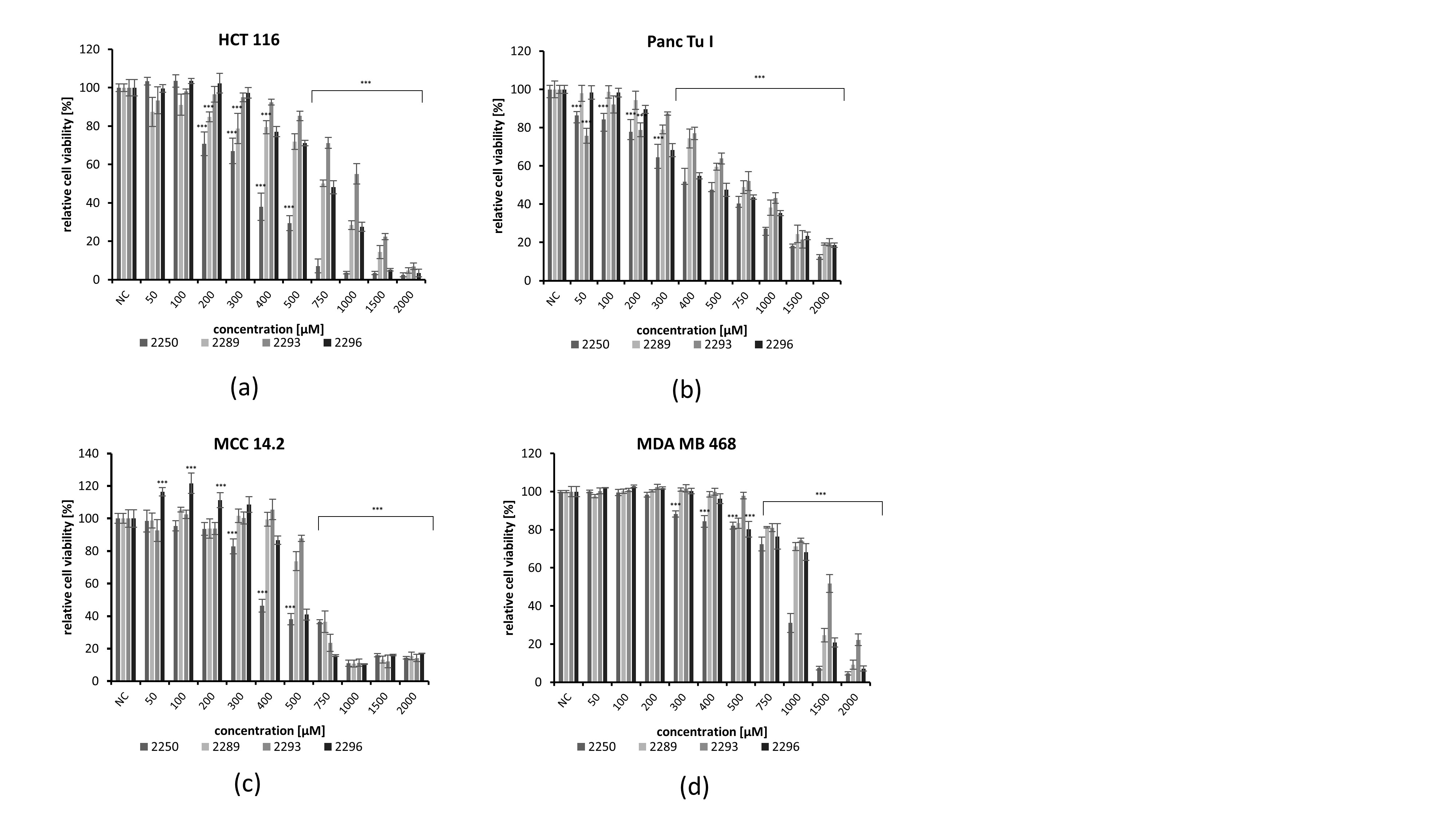

Supplement: Supplementary file 1 — Supplementary file1 (JPG 571 KB) [file 432_2023_4799_MOESM1_ESM.jpg]

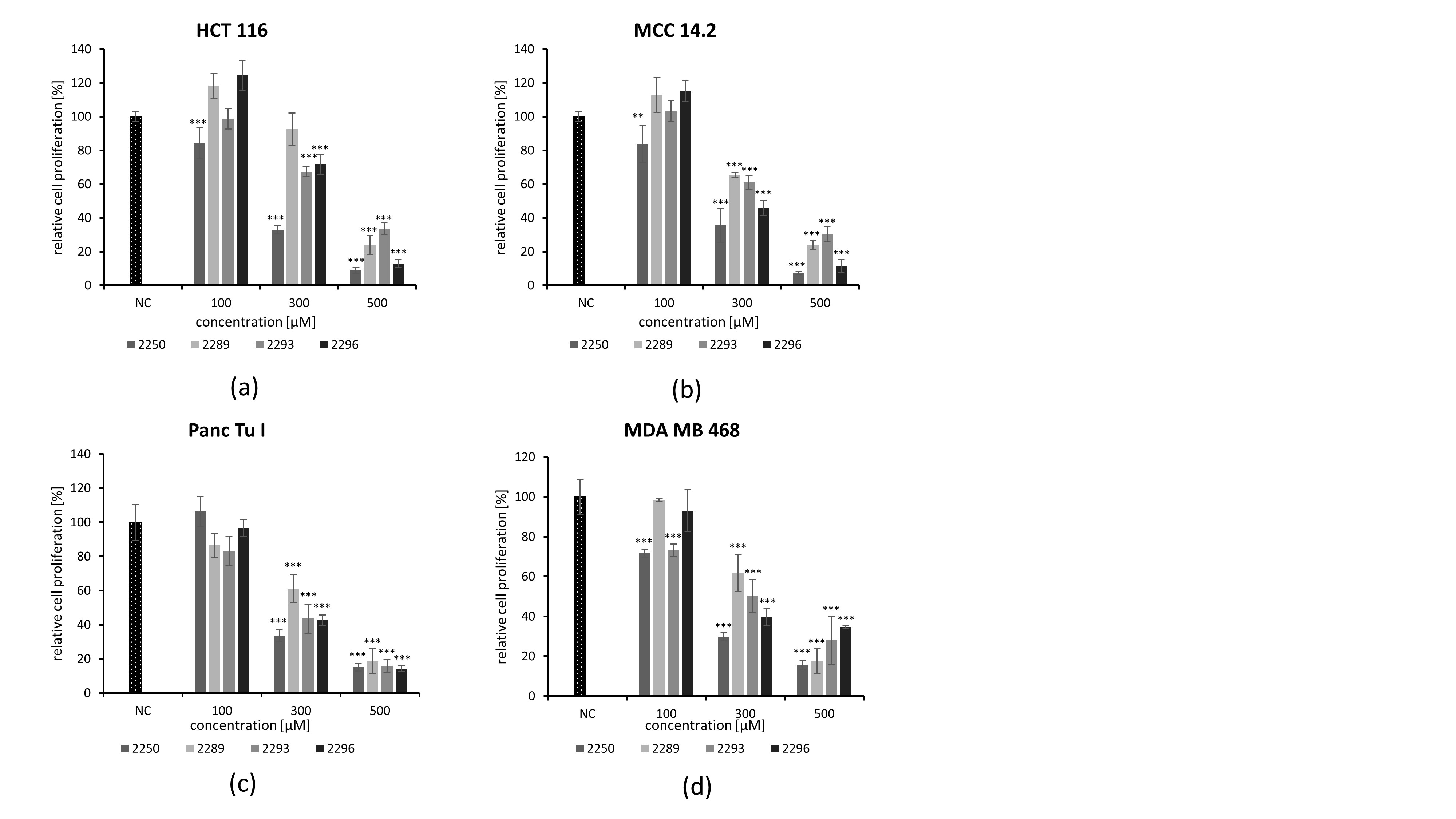

Supplement: Supplementary file 2 — Supplementary file2 (JPG 442 KB) [file 432_2023_4799_MOESM2_ESM.jpg]

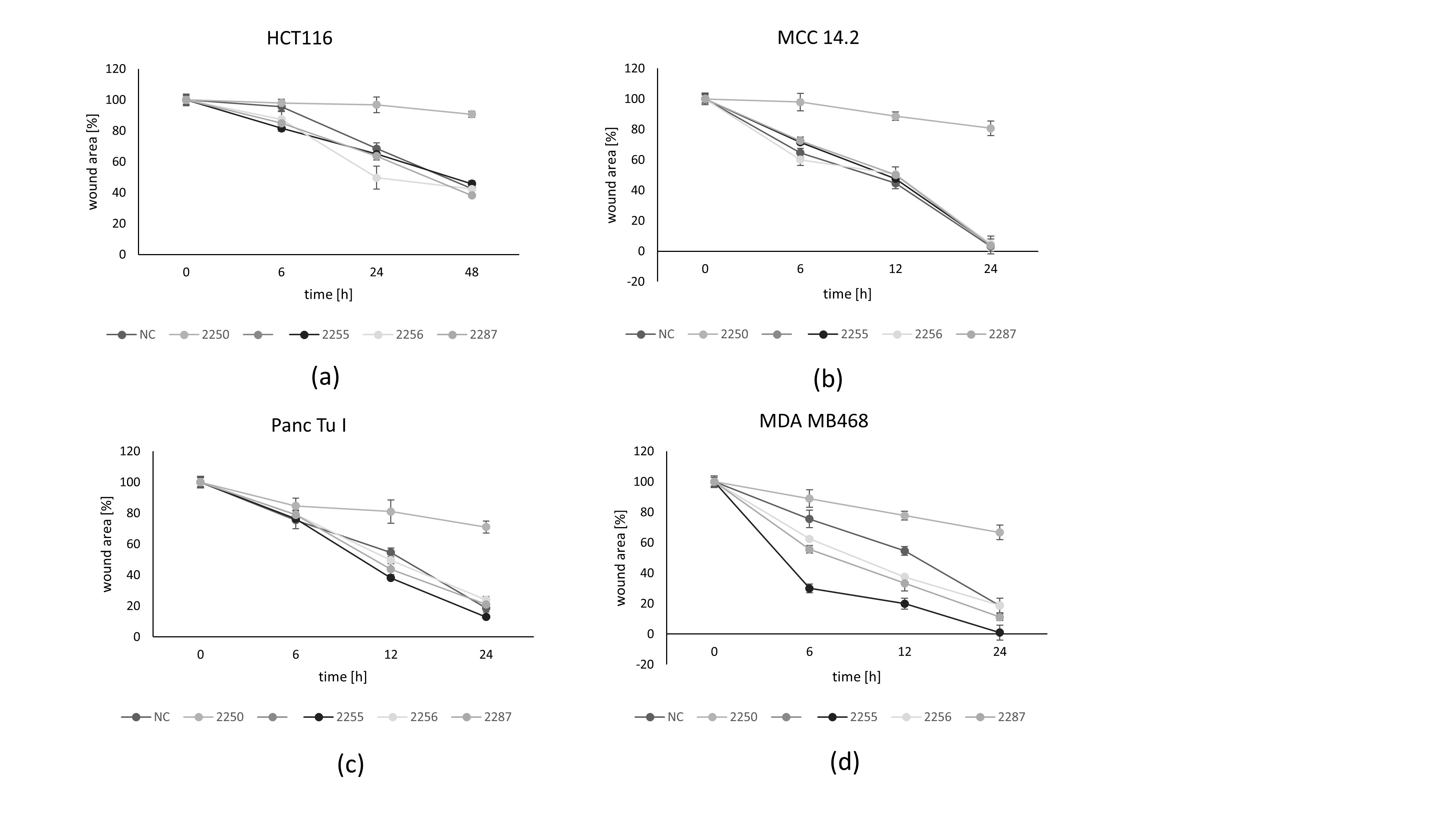

Supplement: Supplementary file 3 — Supplementary file3 (JPG 359 KB) [file 432_2023_4799_MOESM3_ESM.jpg]
